# Unmet needs for care for activities of daily living among older adults with functional disabilities in Vietnam

**DOI:** 10.3389/fpubh.2023.1216785

**Published:** 2023-10-02

**Authors:** Phong Manh Phi, Long Thanh Giang, Tham Thi Hong Pham

**Affiliations:** ^1^Faculty of Political Studies, Hanoi University of Mining and Geology, Hanoi, Vietnam; ^2^Faculty of Economics, National Economics University, Hanoi, Vietnam; ^3^Faculty of Mathematical Economics, National Economics University, Hanoi, Vietnam

**Keywords:** activities of daily living (ADLs), aging, care, older persons, unmet needs, Vietnam

## Abstract

**Background:**

Given its low-middle-income status, Vietnam is experiencing a rapidly aging population. Along with this demographic trend, the care needs of older adults, particularly those with functional disabilities, have become an emerging policy issue.

**Purpose:**

This study examined the prevalence of unmet needs for care in activities of daily living (ADLs) among Vietnamese older adults with functional disabilities.

**Methods:**

We used data from the Population Change and Family Planning Survey (PCS) in 2021, which was a nationally representative survey. Cross-tabulations and logistic regressions were applied to identify older adults' individual and household factors associated with their unmet care needs.

**Results:**

Overall, 4.80% of older adults with at least one functional disability needing care to perform one or more ADLs suffered from unmet needs, of whom 2.32% did not receive any care and 3.05% received insufficient assistance. Logistic regression results revealed that age, sex, place of residence, ethnicity, marital status, education levels, and self-rated health were significantly associated with unmet needs. The higher risk of having unmet needs is associated with those in middle age (70–79), men, rural residents, ethnic minorities, currently unmarried people, those with less than a primary educational level, and those with normal or poor self-rated health.

**Conclusion:**

Attention should be paid to vulnerable older adults, such as those living in rural areas with poor health status, in order to reduce their unmet needs for ADL assistance.

## Introduction

Population aging, which has resulted from declining fertility rates and increasing life expectancies, is considered an important demographic trend in the 21st century [United Nations Economic and Social Commission for Asia and the Pacific, (1)]. The United Nations Department for Economic and Social Affairs (2) showed that the world's older population (defined as those aged 60 years and above) is expected to increase from 13% in 2019 to ~25% in 2050, and approximately two-thirds of this population would live in low- and middle-income countries (LMICs). As aging is strongly related to increasing health problems and thus higher risks of disability and chronic diseases, such a demographic trend results in higher care needs for older adults (3–5). Due to underdeveloped healthcare delivery systems and care-related policies for older adults in LMICs, such demographic aging may generate various challenges in aged care (4, 6). An unmet need for care, particularly among vulnerable older adults, has emerged in policy considerations (7, 8).

As a middle-income country, Vietnam is experiencing a rapid demographic shift from an aging to an aged society. The Vietnamese population was 98.28 million in 2021, and the older population (those aged 60 years and above) was 12.58 million, accounting for 12.80% of the total population (9). The Vietnamese older population is expected to reach 17.28 million in 2029, 22.29 million in 2039, and 28.61 million in 2049, respectively, accounting for 16.53, 20.21, and 24.88% of the total population (10). One of the rising challenges resulting from an aging population is to meet the care needs of older adults.

On the need side, a rapidly aging population leads to a speedy increase in the number and share of older adults, especially those at the oldest ages, and an increasing burden of disease as a result of non-communicable diseases and functional disabilities among older adults [the Ministry of Health and Health Partnership Group (11)]. There were 1.47 million older adults, accounting for 11.7% of the older population, who suffered at least one functional disability (i.e., those who self-assessed as “very difficult” or “could not perform” one of the following functions: seeing; hearing; mobility; cognition; and communication) (9). Older adults with functional disabilities for a prolonged period may face increased difficulties in performing activities of daily living (ADLs), and they need care from other people to fulfill ADLs. Their care needs might be higher than those without functional disabilities. The number of older adults with functional disabilities is expected to increase from nearly 1 million in 2019 to 2.5 million by 2049 if there is no change in the prevalence of disability in terms of age group (11).

On the provision side, based on the traditional culture of intergenerational relationships, most of the care provision for older adults came from their family members, while other sources of care (such as hospitals and other institutions) were limited (11). Approximately 97% of the care received by Vietnamese older adults was provided by family members, and the main care providers were spouses, children, and grandchildren (12). Nevertheless, such traditional family care has been decreasing due to Vietnam's recent sociodemographic changes: smaller household size, children working away from home for better employment, an increasing labor force participation rate among women who have played a primarily home-based care role for older adults, and an increasing number of older adults living alone (particularly the oldest - those aged 80 years and above), living with their spouse only, or living in skip-generation households. The main source of care for older adults is still family members because the development of a long-term care system and public social assistance programs for older adults is at an initial stage in Vietnam (11, 13).

In terms of unmet need, it has been conceptualized as the gap between the amount of long-term care needed as assessed by an individual and the actual resources an individual has at their disposal to meet that need (14). In other words, a person who needs care but whose care provision is unavailable or insufficient is considered to have an unmet need (15). The unmet need could cause a range of adverse consequences, such as more doctor visits, hospitalizations, poor self-assessed health, poor quality of life, higher risks of institutionalization and death (16), more severe disability (17), and an increasing risk of falls (18, 19).

To mitigate such negative consequences and improve the quality of life for the older population, it is important to identify the prevalence and factors associated with the unmet need for personal assistance with ADLs. Given this crucial phase of an aging population, a limited number of studies have been performed to address this issue in Vietnam. To date, few studies have investigated the unmet need for ADL assistance among Vietnamese people aged 60 years and above by using data from the FilaBavi Demographic Surveillance System. For example, Hoi et al. (20) found that a wide proportion of older adults did not receive enough support for different types of ADLs, from 5.1% (for toilet use) to 14.1% (for transferring). Phi et al. (7) found that ~16% of older adults receiving care experienced insufficient care, and 25% of older adults did not receive any care when needed. The Ministry of Health (21) revealed that 3.3% of older adults faced insufficient care.

To our best knowledge, no study has investigated the prevalence and its association with underlying factors of unmet needs among Vietnamese older adults with functional disabilities. As such, this study, using data from a national survey, namely the Population Change and Family Planning Survey 2021 (or PCS 2021), filled such a research gap by providing analyses on unmet needs and their associated factors for Vietnamese older adults with functional disabilities to provide evidence-based policy discussion to the Government of Vietnam.

## Materials and methods

### Materials

This study used the data from the Vietnamese Population Change and Family Planning Survey (PCS) in 2021, which was conducted under Decision No. 1903/QD-TCTK dated 30 December 2020 by the GSO Director General. PCS is an annual survey collecting information about the Vietnamese population, comprising basic characteristics of the population, population changes, and the extent to which family planning methods have been used. A total of 1,083,160 participants were recruited for the final survey sample.

For the first time, a new module on older adults and their issues was included in PCS 2021. In the final sample of PCS 2021, there were 148,413 older adults (defined as those aged 60 years and above). A variety of information on older adults was included in the PCS 2021, such as self-rated health status, functional disabilities, activities of daily living (ADLs), and care receipts. These sets of information have been standardized and widely employed by international and local organizations (such as GSO, VWU, and UNFPA) in sociodemographic and health surveys that allowed us to answer the research questions of this study. Particularly, for disability measures, PCS 2021 applied the Washington Group Short Set on Functioning Questions (22), which was also used in the first national survey on disabilities in Vietnam in 2016 (23).

### Data analysis

Data analysis was conducted using the Stata 14.1 software package (College Station, Texas, 77845, USA). A one-way ANOVA test was employed to examine the association between unmet needs and independent variables. A multivariable logistic regression analysis was used to identify significant risk factors for unmet needs.

In all calculations, we used the sample weight to have representative results for all older adults and their sub-groups (such as those by age, sex, ethnicity, and place of residence).

### Variable measures

#### Measure of functional disabilities

The PCS 2021 incorporated questions to collect a variety of information to assess older adults' functional disabilities. Older adults were asked about their functional abilities, including (i) seeing (even with glasses), (ii) hearing (even with a hearing aid), (iii) mobility (walking or climbing a staircase), (iv) cognition (remembering or concentrating); and (v) communicating in a common language (understanding or being understood). They were asked to self-assess the difficulty levels when performing these functions by selecting one of the following answers: (i) not difficult at all, (ii) a bit difficult, (iii) very difficult, and (iv) could not perform.

In this study, we applied the same definition of a disabled person as that outlined in GSO (23) for an older person, i.e., an older person was considered to have a disability in a function if they chose “very difficult” or “could not perform” for that function. An older person was regarded as having at least one functional disability if they had one or more of the functional disabilities listed above.

#### Measure of care need

A crucial factor influencing the care needs of older adults was how well they could perform activities of daily living (ADLs), including (i) eating, (ii) putting on and taking off clothes, (iii) bathing and washing, (iv) getting up when lying down; and (v) getting to and using the toilet. The PCS 2021 included questions to ask for information on older adults' difficulty levels in ADLs. They were asked to self-assess the difficulty levels when performing the ADLs mentioned above by selecting one of the following answers: (i) not difficult at all; (ii) a bit difficult; (iii) very difficult; and (iv) could not perform.

This study defined that an older person needed care if they were self-assessed to be very difficult or could not perform any of the above ADLs. This definition of care need is consistent with that used in GSO et al. (9).

#### Measures of unmet needs for care

**Figure 1:**
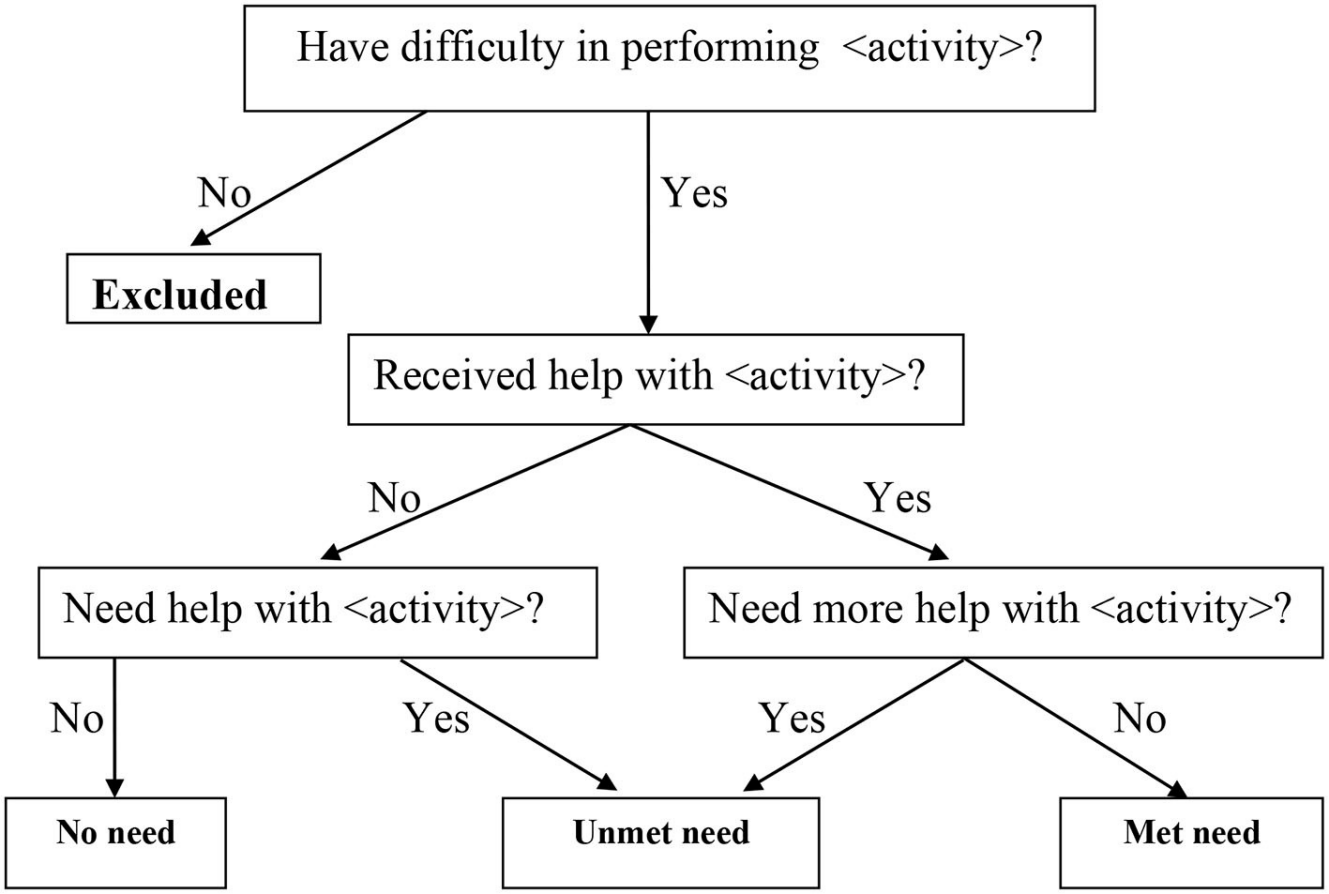
Determination of unmet care need. Source: Desai et al. (24).

Figure 1 illustrates unmet needs for care among older adults. Two indicators of unmet needs were analyzed.

The first indicator (“unmet type 1”) showed a situation where an older person needed personal assistance in ADLs from others, and they received it, but it was insufficient. It was a dichotomous variable describing whether or not the care recipient reported that their personal care was adequate.

The second indicator (“unmet type 2”) showed a situation where an older person needed personal assistance in ADLs from others but did not receive it. This type was also measured as a dichotomous variable, demonstrating whether or not the respondent needed personal care.

A person was considered to have an unmet need if they experienced “unmet type 1” or “unmet type 2”. The PCS 2021 contained full information on care receipts and whether they were enough for a receiver, allowing us to determine who had an unmet need.

#### Independent variables

In this research, two categories of independent variables present the sociodemographic and health characteristics of older adults.

Sociodemographic variables included age, sex, place of residence, ethnicity, marital status, and the highest education level. *Age* was divided into three sub-groups (60–69, 70–79, and 80 years and over). *Sex* was a binary variable identifying whether the respondent was male or female. *Place of residence* was a dichotomous variable confirming whether the respondent was living in a rural or urban area at the time of the survey. *Ethnicity* was a binary variable to identify whether the respondent was Kinh (Vietnamese majority) or non-Kinh (other ethnic minorities). *Marital status* was divided into three sub-groups: currently married, widowed, and other statuses (single, divorced, or separated). *The highest education level* was measured as a binary variable showing whether the respondent had less than the primary level (i.e., never schooling or incomplete primary education) or completed primary education or above.

Health characteristics were presented by self-rated health status, which was divided into three sub-groups to reveal whether the respondent was in poor, normal, or good health status.

## Results

### Sample characteristics

Table 1 shows that 50.59% of Vietnamese older adults have at least one functional disability. In terms of characteristics, the one-way ANOVA test revealed statistically significant differences between care needs and sociodemographic and health statuses. The rate of older adults needing care increased with more advanced age groups (38.98, 42.41, and 60.59% for 60–69, 70–79, and 80 and over, respectively, *p* < 0.0001). Older women had higher care needs than older men (51.52 vs. 48.97%, *p* < 0.0001). Urban older adults reported a slightly higher care needs rate than their rural counterparts (51.58 vs. 50.11%, *p* < 0.05). Kinh people had a significantly higher rate of care needs than their non-Kinh counterparts (50.97 vs. 47.70%, *p* < 0.0001). Older adults with widowed status had the highest prevalence of care needs (55.44%), followed by the currently married group (46.24%) and the other (single, separated, or divorced) (42.42%, *p* < 0.0001). The percentage of older adults with less than a primary education level experienced higher care needs than those with a primary or above education level (53.37 vs. 48.99%, *p* < 0.0001). Older adults with worse self-rated health status had higher care needs than those with better status: 18.04, 20.40, and 59.74% for good, normal, and poor health status, respectively (*p* < 0.0001).

**Table 1:** Prevalence of care needs among older adults with at least one functional disability.

**Characteristics**	**1**+ **ADL disabilities** ***n*** = **18,409**	
	**%**	***P*** **-value**
**Total**	**50.59**	
Age		0.0000
60–69	38.98	
70–79	42.41	
80+	60.59	
Sex		0.0000
Male	48.97	
Female	51.52	
Residence		0.0404
Urban	51.58	
Rural	50.11	
Ethnicity		0.0000
Kinh	50.97	
Non-Kinh	47.70	
Marital status		0.0000
Currently married	46.24	
Widowed	55.44	
Other (single, divorced, separated)	42.42	
The highest education level		0.000
Less than primary	53.37	
Primary or above	48.99	
Self-rated health status		0.0000
Good	18.04	
Normal	20.40	
Poor	59.74	

Source: Own calculations, using data from PCS 2021.

### Factors associated with the care needs of older adults with functional disabilities

Table 2 describes the results of the logistic regression model in which sociodemographic and health variables were used to predict the probability of the need for ADL assistance among older adults with functional disabilities.

**Table 2:** Odds ratios and two-tailed *p*-value from binary logistic regression models predicting care needs among older adults with at least one functional disability.

**Variables**	**Odds ratios (*n* = 18,409)**	***P*-value**
**Age**		
60–69 (ref.)		
70–79	1.059	0.000
80+	1.984	0.000
**Sex**		
Female (ref.)		
Male	1.081	0.000
**Residence**		
Rural (ref.)		
Urban	1.132	0.000
**Ethnicity**		
Non-Kinh (ref)		
Kinh	1.062	0.000
**Marital status**		
Currently married (ref.)		
Widowed	1.101	0.000
Other statuses	1.064	0.000
**Education levels**		
Primary or above (ref.)		
Less than primary	1.026	0.000
**Self-rated health status**		
Good (ref.)		
Normal	1.098	0.000
Poor	5.943	0.000

Source: Own calculations, using data from PCS 2021.

The results show that the likelihood of needing care increased with more advanced age (OR = 1.059 for 70–79; OR = 1.984 for 80 and over, *p* < 0.0001), men (OR = 1.081, *p* < 0.0001), urban residents (OR = 1.132, *p* < 0.0001), Kinh people (OR = 1.062, *p* < 0.0001), those who were widowed or in other marital statuses (OR = 1.101 for the widowed; OR = 1.064 for the other statuses, *p* < 0.0001), those with less than primary education level (OR = 1.026, *p* < 0.0001), and those with worse self-rated health status (OR = 1.098 for the normal health; OR = 5.943 for the bad health, *p* < 0.0001).

### An unmet need for care and its associated factors among older adults with functional disabilities

The rates of unmet need for ADL assistance are presented in Table 3. The overall rate of unmet needs for ADLs among older adults with at least one functional disability was 4.08%, in which the percentages of “unmet need type 1” (received care but not enough as expected) and “unmet type 2” (did not receive any care when needed) were 3.05 and 2.32%, respectively.

**Table 3:** Prevalence rates of unmet needs among older adults with at least one functional disability.

**Characteristics**	**Type 1 (%) *n* = 7,621**	***P*-value**	**Type 2 (%) *n* = 9,387**	***P*-value**	**Total (%) *n* = 9,387**	***P*-value**
Total	3.05		2.32		4.80	
Age		0.0095		0.4382		0.1648
60–69	4.04		1.83		4.83	
70–79	3.52		2.71		5.49	
80+	2.61		2.33		4.53	
Sex		0.6536		0.6855		0.8904
Male	3.15		2.42		5.00	
Female	2.99		2.27		4.69	
Residence		0.2174		0.0182		0.0107
Urban	2.21		2.00		3.75	
Rural	3.45		2.48		5.32	
Ethnicity		0.0129		0.6723		0.0570
Kinh	2.91		2.28		4.66	
Non-Kinh	4.19		2.62		5.92	
Marital status		0.0002		0.0075		0.0000
Currently married	3.08		1.98		4.43	
Widowed	2.79		2.45		4.78	
Other statuses	6.41		4.02		8.47	
Education levels		0.1366		0.6810		0.4312
Less than primary	3.54		2.21		5.11	
Primary or above	2.73		2.39		4.61 |	
Self-rated health status		0.3535		0.3470		0.9164
Good	1.59		2.37		3.38	
Normal	2.34		2.62		4.24	
Poor	3.11		2.29		4.87	

Source: Own calculations, using data from PCS 2021.

When examining sociodemographic and health characteristics, the one-way ANOVA tests indicated statistically significant differences between unmet care needs and some characteristics. More advanced age groups were associated with a lower rate of unmet need type 1 (4.04%; 3.52%; 2.61% for 60–69; 70–79; 80 and over, respectively, *p* < 0.01). Rural residents had a higher rate of “unmet need type 2” compared to their urban counterparts (2.48 vs. 2.00%, *p* < 0.05). The percentage of Kinh people had a lower percentage of “unmet need type 1” than their counterparts (2.91 vs. 4.19%, *p* < 0.05). Currently married people had the lowest rate of “unmet need type 1” and “unmet need type 2” (3.08 and 1.98%, respectively), followed by widowed people (2.79 and 2.45%, respectively), and the highest rate was for those with other marital statuses (6.41 and 4.02%, respectively) (*p* < 0.01). However, the prevalence of “unmet need type 1” did not significantly vary by sex, residence, the highest education level, or self-rated health status. For “unmet need type 2”, age, sex, ethnicity, the highest education level, or self-rated health status did not significantly vary.

In terms of general unmet need status (by combining “unmet need type 1” and “unmet need type 2”), the results showed that rural residents had statistically significantly higher rates than their urban counterparts (5.32 vs. 3.75%, *p* < 0.05). For marital status, older adults with other statuses (single, separated, or divorced) had a significantly higher unmet need rate than those who were currently married or widowed (8.47 vs. 4.43 and 4.78%, respectively, *p* < 0.0001). In contrast, the one-way ANOVA tests indicated that the prevalence of unmet needs insignificantly varies by age, sex, ethnicity, the highest education level, and self-rated health status.

A logistic regression was employed to determine factors associated with the unmet need for ADL assistance among older adults with functional disabilities. The results are presented in Table 4. The likelihood of having unmet needs increased among middle-aged people (70–79) (OR = 1.197, *p* < 0.001); men (OR = 1.189, *p* < 0.001); those who were widowed or had other marital statuses (OR = 1.186 and OR = 2.241, respectively, *p* < 0.001); those with less than primary education level (OR = 1.137, *p* < 0.001), and those with worse self-rated health status (OR = 1.250 for those with normal health; OR = 1.458 for those with poor health, *p* < 0.001), compared with their respective reference groups.

**Table 4:** Odds ratios and two-tailed *p*-value from binary logistic regression models predicting unmet needs among older adults with at least one functional disability.

**Variables**	**Odds ratios—OR (*n* = 9,387)**	***P*-value**
**Age**		
60–69 (ref.)		
70–79	1.197	0.000
80+	0.956	0.006
**Sex**		
Female (ref.)		
Male	1.189	0.000
**Residence**		
Rural (ref.)		
Urban	0.701	0.000
**Ethnicity**		
Non-Kinh (ref.)		
Kinh	0.812	0.000
**Marital status**		
Currently married (ref.)		
Widowed	1.186	0.000
Other statuses	2.241	0.000
**Education levels**		
Primary or above (ref.)		
Less than primary	1.137	0.000
**Self-rated health status**		
Good (ref.)		
Normal	1.250	0.000
Poor	1.458	0.000

Source: Own calculations, using data from PCS 2021.

In contrast, the likelihood of having unmet care needs decreased among the “oldest old” (those aged 80 years and above) (OR = 0.956, *p* < 0.01), urban residents (OR = 0.701, *p* < 0.001), and Kinh people (OR = 0.812, *p* < 0.001).

## Discussion

The first aim of this study was to investigate the prevalence of the unmet need for ADL assistance among older Vietnamese people with functional disabilities. Using the data from the PCS 2021, we found that 4.80% of older adults reported having unmet needs, in which 3.05% received assistance but not as expected (“unmet need type 1”) and 2.32% did not receive any care (“unmet need type 2”). Yet, it is hard to compare estimates of unmet need for ADL across studies because of considerable differences in the study methods, the sample characteristics, and the definitions of disability, care need, and unmet care need (24). For example, this study identified an older person needing care if they self-assessed as “very difficult to perform ADLs” or “unable to perform ADLs,” while Phi et al. (7) defined a person with a need for ADL assistance if they had ADL difficulty at any level. Moreover, our study examined the status of unmet needs among older adults with functional disabilities instead of the entire older population.

Our estimates might be similar to the prevalence rate previously reported by MOH et al. (21), where 3.3% of older adults suffered from “unmet need type 1”. However, the rate of unmet needs obtained in this study might be relatively low compared to other studies conducted in Vietnam. For example, the rate of Vietnamese older adults who did not receive enough care for ADLs varied from 5.1 to 14.1% for specific ADLs (20). By using data from the 2011 Vietnam Aging Survey, Phi et al. (7) found that around 16% of older Vietnamese received inadequate support, and 25% of those did not receive any care when needed. Using data from a cross-sectional survey of 695 individuals aged 60 years and older conducted in Thanh Hoa City and Hoang Hoa district (Vietnam), Nguyen and Assanangkornchai (25) found that 11.6% of the respondents reported difficulties in performing basic ADLs. Individuals with worse self-reported health, two or more chronic diseases, hearing impairment, cognitive impairment, unemployment, and living in rural areas were more likely to be disabled in basic ADLs than their counterparts. Vu et al. (26), examining 412 patients aged 60 years and older with type 2 diabetes mellitus, showed that 79.4% of those had depressive symptoms, and the increased likelihood of having depressive symptoms was related to an impairment of IADLs. Utilizing cross-sectional data with 251 individuals aged 80 years and older in Soc Son district (Vietnam), Nguyen et al. (27) showed that 11.2% of them were considered frail, and 64.5% of them had three or more IADL impairments. Among the frailty components, low walking speed and low physical activity were significantly associated with an increased likelihood of having three or more IADL impairments (which was considered a functional disability). Nguyen et al. (28) indicated that patients who were in the American Spinal Cord Injury Association (ASIA) scale group A (complete dysfunction) had the lowest ADL and IADL index.

In Asian countries, there have been few studies specifically examining this issue. For example, using data from a cross-sectional survey of 2,695 community-dwelling older individuals aged 60 years or above living in five rural Asian towns (Indonesia: 411; Vietnam: 379; Japan: 1,905) between June 2002 and July 2003, Wada et al. (29) showed that those with depression had significantly lower scores for each item of the ADL than those without depression in all three countries. Momtaz et al. (19) indicated that 18.3% of functionally disabled older Malaysians (those aged years 60 and above) experienced unmet needs of both ADLs and IADLs (Instrumental Activities of Daily Living). Phetsitong and Vapattanawong (30) showed that rates of Thai older adults in households with an unmet need for caregivers were 14.6% in 2007, 17.5% in 2011, 26.5% in 2014, and 22.9% in 2017, which were observation units of households having at least one older person who needed ADL care. Gu and Vlosky (31) showed that ~60% of the Chinese older population (aged 60 years and above) could not meet their ADL assistance needs. Chen et al. (32) showed that the highest prevalence rate of unmet needs for care for Chinese older adults (aged 60 years and above) ranged from 4.6 to 77.2% for different ADL tasks. Analyzing the data from the 2018 Chinese Longitudinal Healthy Longevity Survey, Cao et al. (33) showed that disabled older adults (65 years and over) experiencing unmet needs had a lower likelihood of obtaining a higher level of healthy aging. Yau et al. (34), using the data from 34 studies conducted in the ASEAN region, performed a systematic literature search to analyze the functional disabilities (ADL and/or IADL disability) status of older adults (aged 60 years and above) and its impact. The result revealed that the pooled rate of ADL disability was 21.5%, and that of IADL disability was 46.8%. Higher rates were for those of more advanced age and women. The increased years of life with disability and poor health-related quality of life had adverse impacts.

Studies carried out in other regions of the world also showed higher percentages of unmet needs. For example, 34.6% of the older population (aged 65 years and above) in the United States with chronic disabilities did not get care to perform ADLs (35). Desai et al. (24) showed that 20.7% of older Americans (aged 70 years and above) needing ADL help had an unmet need. Using the data obtained from the Survey of Health, Aging, and Retirement in Europe, Pego and Nunes (36) indicated that 39.5% of the respondents aged 65 years and above with functional dependence (limited in at least one ADL or IADL) did not receive informal care.

The second aim of this study was to investigate sociodemographic and health characteristics associated with unmet needs among older Vietnamese people with functional disabilities. The results from logistic regression showed that age, sex, residence, ethnicity, marital status, the highest education level, and self-rated health status were significantly associated with unmet needs. Our finding was not consistent with a number of studies reporting an insignificant association between unmet needs and age (19, 24, 31, 37, 38) but was consistent with the finding that the oldest old (those aged years 80 and above) had less likelihood to report an unmet need for care compared to the youngest old (aged 60–64 years) (39).

Our results also indicated that men were ~19% more likely than women to report unmet needs, and this is consistent with the finding from Peng et al. (38). This result, however, was inconsistent with studies reporting that women had a higher risk of unmet needs than men (15, 19, 39) or showing that there was no gender-significant difference (31, 32, 37).

In this study, urban respondents were ~30% less likely to have unmet needs than their rural counterparts, which is similar to the findings of other studies (31, 40). But this finding was in contrast with that of Gibson and Verma (17), who found that urban respondents had higher levels of unmet need than their rural counterparts. In between, an insignificant association between residence and unmet needs was found in other studies (18, 41).

Kinh people had an ~19% lower risk of unmet needs than their counterparts. This finding is similar to the finding from other studies [such as (38)], which found that Han ethnicity was 32% less likely to have unmet needs than non-Han counterparts in the case of China. However, no significant association between race and unmet needs was found in various studies (15, 17, 19, 24, 42).

We found a higher likelihood of unmet needs among older adults who were widowed or in other marital statuses (single, divorced, or separated) than currently married people, particularly those with other marital statuses, who were 2.241 times more likely to experience unmet needs than currently married people. Some previous research examined the correlation between marital status and unmet need for ADL but found an insignificant correlation (15, 19, 31, 32).

Older adults with less than a primary education level had a higher likelihood of unmet needs (1.137 times) than those with a primary or above degree. Other studies found that the highest education level did not have a significant relationship with unmet needs (24, 31, 32, 38, 39, 41, 43, 44).

Compared to those in good health, the risk of unmet needs for those in normal and poor health was higher at 1.250 and 1.458 times, respectively. This finding is consistent with those found in other studies [such as (38, 44)]. However, Otero et al. (43) found that self-rated health was not significantly associated with unmet needs.

## Conclusion

The findings from this study indicated that many older Vietnamese people had functional disabilities. Among these people, those with other marital statuses (single, separated, or divorced) had the lowest rate of care needs but the highest rate of unmet needs. Those living in rural areas and those with poor health status experienced higher unmet need rates than their urban counterparts and those with normal or good health status. It is suggested that the most vulnerable groups need more attention from policymakers in Vietnam to address the gaps in their care needs.

With the rapidly aging population, the care needs of older adults will be rising. However, the traditional care provided by family members is declining due to migration and smaller household sizes, and older adults are generally not affordable for care services in public institutions or private facilities. As such, the development of affordable home-based and community-based care services should be a strategic direction for Vietnam (11).

Although this is the first study to use a nationally representative survey to examine unmet care needs among Vietnamese older adults with functional disabilities, and it adds quantitative evidence to the Asian literature, there are some limitations to consider in future studies. The first limitation is related to the usage of a cross-sectional data set because we could not analyze a causal relationship between dependent and independent variables. The second limitation emerges from the data collected using the self-reporting method because it might underestimate or overestimate the levels of need and unmet needs for care. The third limitation is that, beyond the sociodemographic and health factors of older adults, other related factors concerning household and community characteristics could impact older adults' unmet care needs, but they were not available in the PCS 2021 data. If we could have had comparable data sets to combine with the PCS 2021 data, the propensity score matching (PSM) method could have been applied to further explore the impacts of these factors.

## Data availability statement

The data analyzed in this study is subject to the following licenses/restrictions: data set is not allowed to be publicly shared. Requests to access these datasets should be directed to not available.

## Author contributions

PP formed the research questions, writing ideas, calculated data, and wrote the draft. LG formed the research questions, checked data and calculations, revised, and finalized the draft. TP supported in data processes and calculations. All authors approved the submitted version.
